# Pulmonary metastasis presenting as a ground glass opacity-like lesion with a thin-walled cavity: A case report

**DOI:** 10.1016/j.ijscr.2019.06.030

**Published:** 2019-06-21

**Authors:** Akira Haro, Sho Wakasu, Kazuki Takada, Atsushi Osoegawa, Takeshi Kamitani, Tetsuzo Tagawa, Masaki Mori

**Affiliations:** a Deparment of Surgery and Science, Kyushu University Hospital, Fukuoka, Japan; b Department of Anatomic Pathology, Pathological Science, Kyushu University Hospital, Fukuoka, Japan; c Department of Clinical Radiology, Kyushu University Hospital, Fukuoka, Japan

**Keywords:** Tongue cancer, Pulmonary metastasis, Lymph node metastasis, Thin-walled cavity, Squamous cell carcinoma, Ground glass opacity

## Abstract

**Introduction:**

Most of pulmonary metastases present as well-defined solid and round nodules. Here we report a case of a pulmonary metastasis presenting as a ground glass opacity (GGO)-like lesion with a thin-walled cavity and lymph node metastasis of tongue cancer.

**Case presentaion:**

A 22-year-old man was referred to our department for surgical diagnosis and treatment for a GGO-like pulmonary nodule with a thin-walled cavity in the right lower lobe. He had a history of surgical resection for tongue cancer. The size of the GGO-like lesion with a thin-walled cavity in the center gradually increased. A right lower lobectomy and hilar lymphadenectomy were performed. Postoperative pathology revealed the lesion as pulmonary metastasis and hilar lymph node metastasis of tongue cancer.

**Discussion:**

Our case report of pulmonary metastasis of tongue cancer is rare from the viewpoint of pulmonary GGO-like lesions with a following thin-walled cavity and hilar lymph node metastasis. The positron emission tomography/computed tomography (PET/CT) examination was useful to show right hilar lymph node metastasis.

**Conclusion:**

It is important to make a differential diagnosis of from the pulmonary nodule in case of a GGO-like lesion with a thin-walled cavity.

## Highlights

• Most of pulmonary metastasis present as a well-defined solid and round nodule.
• This is the rare case of a pulmonary metastasis presenting as a ground glass opacity-like lesion with a thin-walled cavity of tongue cancer.
• The positron emission tomography/computed tomography examination was useful to show hilar lymph node metastasis.

## 1 Introduction

Lung is one of the most common metastatic site of malignant solid tumors. Most of pulmonary metastases present as well-defined solid and round nodules, and pulmonary metastasis showing GGO with a thin-walled cavity is rare. Here we present a case of GGO-like pulmonary metastasis with a following thin-walled cavity and lymph node metastasis deriving from primary tongue cancer.

The work has been reported in line with the SCARE criteria and cite the following paper [1].

## 2 Presentation of case

**Fig. 1:**
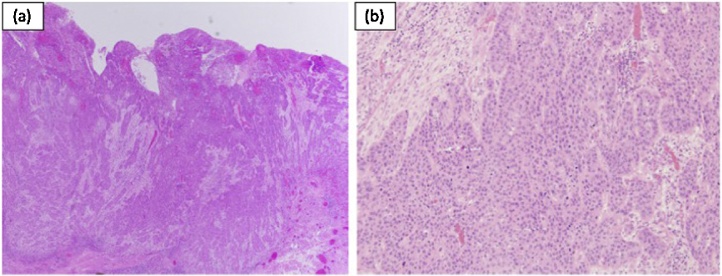
Hematoxylin-eosin staining. **a** Low-power field; primary tongue squamous cell carcinoma, **b** High-power field.

**Fig. 2:**
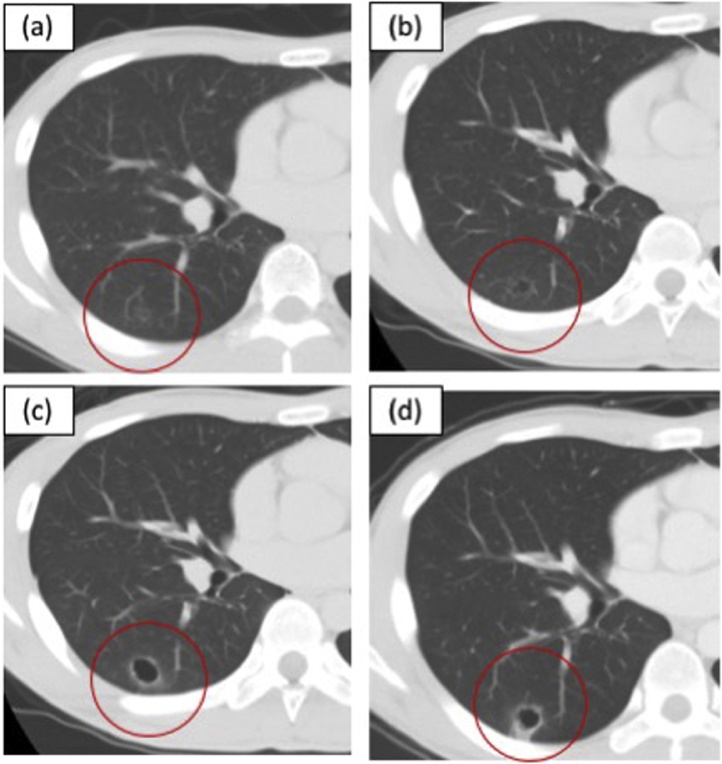
**a** CT showing a ground glass opacity-lesion in the right S6 segment. **b** A thin-walled cavity appeared in the center of the lesion. **c** The cavity extended, and its wall thickened. **d** The GGO-like lesion with the cavity gradually increased in size.

A 22-year-old man was referred to our department for a surgical diagnosis and treatment for a pulmonary lesion. He had no symptoms and his physical examination was normal. Laboratory findings were unremarkable. He was a never-smoker. In his past medical history, he received surgical treatment for primary tongue cancer, at the age of 19 years, and the pathological diagnosis was squamous cell carcinoma (Fig. 1). He subsequently received chemoradiation therapy and surgical resection for cervical lymph node metastasis at the age of 20 years. He had postoperative medical follow-up after those treatments. A small GGO was detected in the right S6 segment 18 months after surgical treatment for primary tongue cancer (Fig. 2a). Subsequently, a thin-walled cavity appeared in the center of the lesion (Fig. 2b) and extended (Fig. 2c). The size of the GGO-like lesion increased from 0.78 cm to 2.37 cm in the chest CT over eight months (Fig. 2d).

**Fig. 3:**
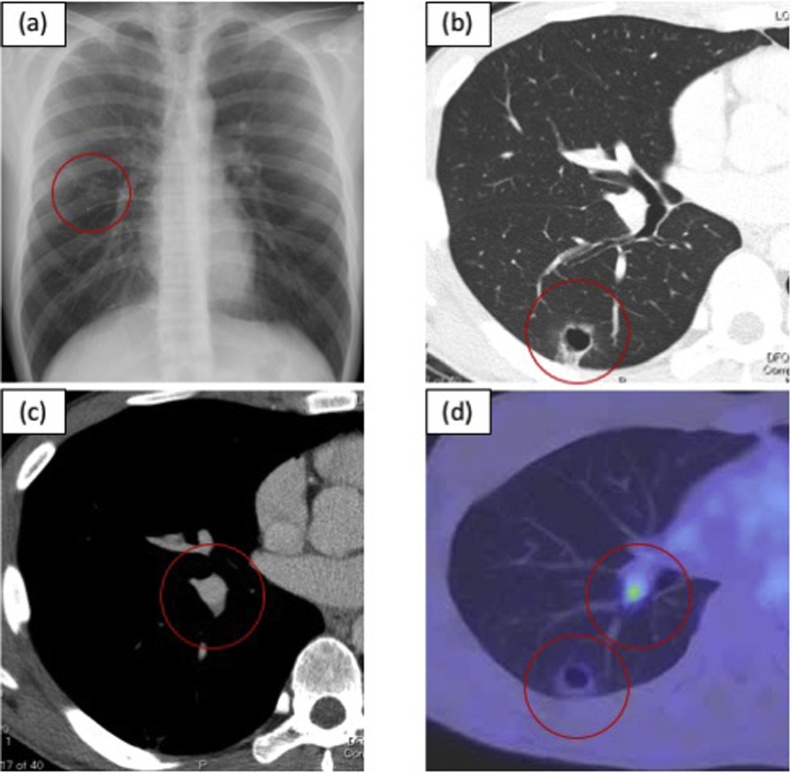
**a** Chest X-ray showed a cavity-lesion in the right middle field. **b** CT showing a GGO-like lesion with a thin-walled cavity. **c** Contrast chest CT showing no finding of an abnormal hilar lymph node metastasis. **d** PET examination showing SUV up take in the lesion with the thin-walled cavity and hilar lymph node.

He underwent a careful examination at our department, and a chest X-ray revealed a pulmonary lesion (Fig. 3a) with no solid component. The wall of the cavity lesion gradually thickened (Fig. 3b). Lymphadenopathy was not detected in the chest CT examination (Fig. 3c), but suspected from the high FDG up take in the PET examination (Fig. 3d). The maximum standardized uptake values for the GGO-like lesion and right hilarity lymph node were 1.41 and 2.94, respectively. There was no abnormal uptake in no other lymph node metastasis or distant metastasis in the PET examination.

**Fig. 4:**
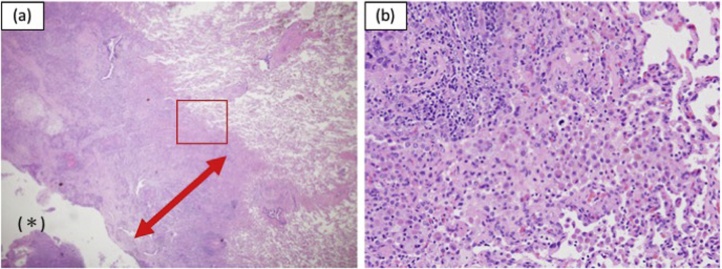
Hematoxylin-eosin staining. **a** Low-power field; cavity (asterisk) and thick-wall (red double-headed arrow) **b** High-power field of the red square of Fig. 4a.

For diagnosis and surgical treatment, a wedge resection of the right lower lobe (right S6 segment) was performed. Since intraoperative histological diagnosis was squamous cell carcinoma, a right lower lobectomy and hilar lymphadenectomy were performed. The patient had an uneventful recovery and was discharged on postoperative day 7. Postoperative pathological diagnosis was pulmonary metastasis (Fig. 4a) and hilar lymph node metastasis deriving from primary tongue cancer. The cancer cells were invading and destroying the alveolar walls, with a surrounding pulmonary hemorrhage (Fig. 4b). Immunohistochemically, carcinoma cells were positive for p40 and p63 (data not shown). He has been receiving adjuvant chemotherapy of S-1 without recurrence for eight months following surgery.

## 3 Discussion

In most cases, pulmonary metastasis is pointed in chest X-ray or chest CT with no symptoms, and it is difficult to make a differential diagnosis of primary lung cancer or a metastatic lung tumor in patients with a history of solid malignant disease. Most of pulmonary metastatic tumors present as well-defined solid and round nodules. Pulmonary metastasis presenting as a GGO-like lesion with a following thin-walled cavity is rare.

GGO-like metastases from some malignant tumors such as breast cancer [2] or malignant melanoma [3] are rarely reported. The microscopic findings of GGO shows a proliferation of metastatic cells from breast cancer or a malignant melanoma in a lepidic-like fashion.

However, a thin-walled lesion is sometimes observed in primary lung adenocarcinoma [4] or angiosarcoma [5], but rarely in lung squamous cell carcinoma [6]. There have been a few reports of a secondary pneumothorax caused by pulmonary metastasis of tongue cancer, in which case the lesions present as a cystic nodule with a cavity [6]. Our case report of pulmonary metastasis of tongue cancer is extremely rare from the viewpoint of pulmonary GGO-like lesions with a following thin-walled cavity and hilar lymph node metastasis.

A metastasectomy is generally selected for treatment of pulmonary metastasis of malignant solid tumors. However, Mochizuki did not recommend a surgical metastasectomy for pulmonary metastasis of tongue cancer because the short interval to the next recurrence and the median survival after the first surgical resection were 4.1 and 9.5 months, respectively [3]. He reported that in addition to wedge resection, pneumonectomy, three bilobectomy, lobectomy and segmentectomy were selected as surgical procedures for complete resection. In his report, a mediastinal and hilar lymphadenectomy was performed in addition to surgical resection of the pulmonary metastasis in some cases; there was lymph node metastasis in 47% of all cases, and particularly hilar lymph node metastasis in 11% of all cases. He reported one case of an incomplete metastasectomy because of the metastatic lymph nodes. In his report, after resection of solitary pulmonary metastasis recurrence occurred in lung, pleural cavity, bone, bone, liver, cervical lymph node, mediastinal lymph node or multiple sites. In this case, pulmonary metastasis and hilar lymph node metastasis occurred with regional lymph node metastasis. According to this, pulmonary metastasis and hilar lymph node metastasis occurred through the process of homogenous metastasis. We first thought to select wedge resection of the right lower lobe (S6 segment), but we decided to proceed with a lobectomy and lymphadenectomy for complete resection of the lymph node metastasis. The PET/CT examination was useful to show right hilar lymph node metastasis.

## 4 Conclusions

We have here reported a rare case of pulmonary metastasis of tongue cancer, presenting as a GGO-like lesion with a following thin-walled cavity and lymph node metastasis.

## Conflicts of interest

All authors (AH, SW, KT, AO, TK, TT & MM) declare no conflicts of interests or disclosures.

## Sources of funding

This work received no funding.

## Ethical approval

The present study was conducted in accordance with the ethical standards of our institution.

## Consent

Informed consent was obtained from the patient for publication of this case report and the accompanying images.

## Author contribution

AH wrote the manuscript. KT, AO, TK and SW supported in the writing of the manuscript. TT and MM supervised the writing of the manuscript. All authors read and approved the final manuscript.

## Registration of research studies

The name of my UIN is researchrestry4858.

## Guarantor

Akira Haro.

## Provenance and peer review

Not commissioned, externally peer-reviewed.
